# Headache and rhinitis: pattern search on Google Trends for 17 years

**DOI:** 10.31744/einstein_journal/2022AO6224

**Published:** 2022-04-25

**Authors:** Diego Belandrino Swerts, Guilherme Barbosa, Mario Fernando Prieto Peres

**Affiliations:** 1 Faculdade Israelita de Ciências da Saúde Albert Einstein Hospital Israelita Albert Einstein São Paulo SP Brazil Faculdade Israelita de Ciências da Saúde Albert Einstein, Hospital Israelita Albert Einstein, São Paulo, SP, Brazil.; 2 Brown University Providence RI United States Brown University, Providence, RI, United States.; 3 Hospital Israelita Albert Einstein São Paulo SP Brazil Hospital Israelita Albert Einstein, São Paulo, SP, Brazil.

**Keywords:** Headache, Rhinitis, Big data, Epidemiology, Alzheimer disease, Migraine disorders

## Abstract

**Objective:**

Headache and rhinitis are highly prevalent and comorbid. The objective of the present study is to analyze the correlation of headache and rhinitis, in addition to the temporal pattern of these diseases in 17 years, using the Google Trends platform.

**Methods:**

Google Trends was searched from January 2004 to June 2021, using the entry: [“*rinite*” (rhinitis) + “*dor de cabeça*” (headache)” + “Alzheimer” + “enxaqueca” (migraine)]. Migraine, primary headache, and Alzheimer’s, with no clear relation with headache, were used as controls. After the descriptive analysis by dispersion diagrams, Pearson’s test and a simple regression model were performed. Subsequently, this study analyzed the seasonality of the volume of research on rhinitis and headache.

**Results:**

A strong correlation between rhinitis and headache (0.86) was found in the time interval analyzed. In addition, a seasonality was identified in the volume of searches for the term rhinitis with increased volume in the fall and peaks in the month of May, with a decrease in the spring and early summer. Moreover, an increase of searches on headache was observed, suggesting worse burden of this pathology.

**Conclusion:**

Headaches and rhinitis were correlated in 17 years of research on the Google Trends platform. Circannual variation of both conditions was observed. Additional studies with digital research may be useful to better understand the epidemiology and comorbidities of headache.

## INTRODUCTION

Healthcare research has been increasingly using search engine data, since they provide valuable information on disease patterns and population behavior.^(1)^Google Trends is a tool to collect this kind of information.^(2)^It is a free online geographical and temporal analysis of the Google Search engine, which receives over three billion daily searches.^(2)^

Google Search data has been used as a research tool in many publications in the medical field, with a wide range of methods and applications. A recent review analyzed 70 articles, covering several topics such as infectious diseases, mental health, non-communicable diseases, and population behavior.^(3)^ Another example of search engine data used for medicinal research was Google Flu Trends.^(4)^ This was a highly impactful tool, created by Google itself, providing data on influenza-like symptom queries in over 25 countries and over ten countries for searches on dengue. The database citations were comparable to the average citations of a scientific paper, with an increase in publications over time.^(5)^

A systematic review analyzed the validation of Google Trends as a research method, and in order to attribute better reproducibility and thereby reliability to the tool, surveys need greater transparency in the methodology. In this way, systematization has given Google Trends greater confidence. No studies with the method in the Brazilian context were found.^(1)^

Headaches and sinus disorders have been linked in several ways. Rhinitis – commonly referred to as hay fever – and chronic headaches are both highly common conditions that coexist in the general world population.^(6)^ Exploring further aspects relating headaches and rhinitis may lead to a better understanding of mechanisms and potential management of these disorders.

Headache may be a symptom of several disorders, or the main aspect in the condition as in primary headaches, such as migraine and tension-type headaches. Primary headaches are very common worldwide and comprise the three most disabling neurological conditions.^(7)^ Comorbidity with psychiatric and other medical conditions often occur, and allergic rhinitis is also frequently associated.

The International Consensus Statement on Allergy and Rhinology defines allergic rhinitis as an immunoglobulin E (IgE)-mediated inflammatory nasal condition resulting from allergen introduction in a sensitized individual. The prevalence of rhinitis varies from 10 to 40% of the population.^(8)^ Allergic rhinitis can result in significant disability, due to its secondary effects in sleep quality, exercise tolerance, fatigue and irritability, depression and cognition, sexual dysfunction, leading to loss of productivity.^(9,10)^

Chronobiology, the branch of biology that studies periodic physiological activities in living organisms, is a topic of intersection between rhinitis and headaches. Although rhinitis has an established seasonal pattern worldwide, and some headaches also present with clear cut seasonal variance, there have been no studies to determine the relationship between these two conditions.

Recent studies show that in many cases the diagnosis of primary headaches is underdiagnosed, leading to drug treatment for allergic rhinitis, causing treatment delay and error, with several associated comorbidities. Understanding the correlation between diseases, their behavior pattern, and the population’s understanding of the topic is important for the correct diagnosis and treatment of diseases.^(11)^

In order to shed light on the association of headache and rhinitis, using a digital epidemiology methodology, the objective of this study is to investigate the correlation between them and their temporal pattern in a search engine database.

## OBJECTIVE

Headache and rhinitis are highly prevalent and comorbid. This study aims to analyze the correlation between headache and rhinitis in Brazil, in addition to the temporal pattern of these diseases in 17 years, using the Google Trends platform.

## METHODS

On June 16^th^, 2021, Google Trends website was searched for the terms “*rinite”* (rhinitis) and *“dor de cabeça”* (headache), limiting the search region to Brazil. As a control and comparison, the terms “*enxaqueca*” (migraine) and Alzheimer’s were also searched. Data were obtained for every month from January 2004 to June 2021, and then extracted to Microsoft Excel file, where the generated numbers were transformed into a graph. The study was conducted at *Faculdade Israelita de Ciências da Saúde Albert Einstein*.

Google Trends data were adjusted proportionately to the time and location of a query in order to make relationships between terms easier to discern. This is accomplished by taking each value for a term and then dividing by the total searches of the geography and time range applicable to that search term. This is done so that places with the most search volume in raw numbers are not always ranked the highest in terms of searches. The results of these calculations were then scaled from 0 to 100 based on the topic proportions to all searches on all topics. This means that different regions which may have the same raw number of searches for a term are not always ranked the same in terms of search volumes.

### Statistical analysis

Data extracted from Google Trends were analyzed comparing the search volume numbers generated by the database. After the descriptive analysis by dispersion diagrams, the Pearson test was performed to evaluate the correlation between the volume of research on rhinitis, headache, and Alzheimer’s disease. The adjusted R-squared was used to assess how close the data are to the regression line, in order to reduce confounding factors. R Statistical program software was then used for the statistical calculations, and p<0.05 was considered statistically significant. A linear regression model, in those with high Pearson correlation, was used to estimate the volume of searches for the term headache and migraine from the term rhinitis, with a 95% confidence interval (CI) to evaluate the prediction of headache research volume from rhinitis research volume.

The Pearson correlation test was performed for the combinations between rhinitis, headache, migraine, and Alzheimer’s. Since there is no correlation between headache or rhinitis with Alzheimer’s, it was used as a control.

Finally, this study analyzed the seasonality of rhinitis and headache research volume. The rhinitis and headache survey volume was issued per month to analyze the seasonal variation of these conditions over the years. Data were normalized and the largest survey volume over the year was considered as 100 percent. As normalization was done per month, the increase in the volume of research on the platform interfered less in results.

## RESULTS

**Figure 1:**
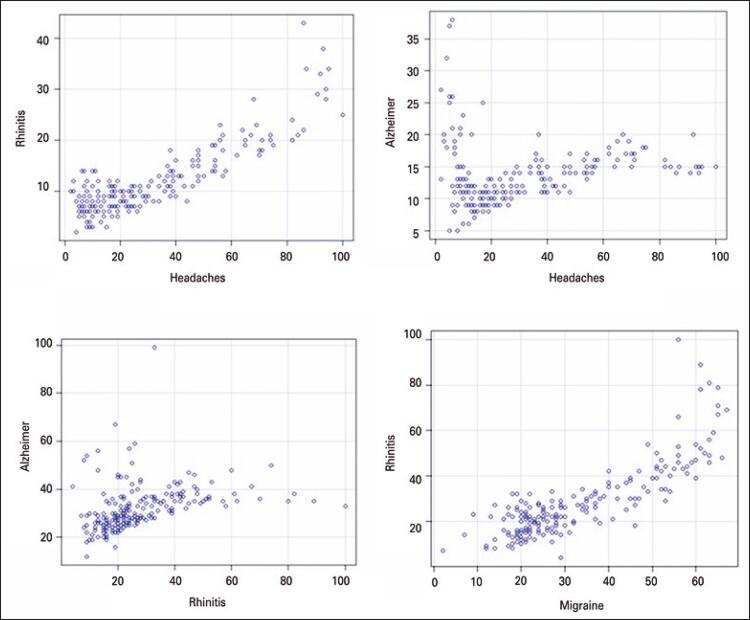
Dispersion diagram

Figure 1 shows the dispersion diagram. The Pearson coefficient for rhinitis and headache was 0.86 (p<0.01), with an adjusted R-squared of 0.75, indicating a strong correlation between the two variables in the time interval analyzed. On the other hand, the test result for headache and rhinitis with Alzheimer’s was respectively 0.14 (p<0.01) and 0.35 (p<0.01), with respectively adjusted R-squared of 0.21 and 0.12, indicating a very low correlation. The correlation between migraine and rhinitis was 0.83 (p<0.01), with adjusted R-squared of 0.69.

The value of p<0.05 in the linear regression test indicates that the linear regression model applied has a good predictor of the variable volume of rhinitis research on the variable headache and migraine search volume. The test result was 3.11, with a 95%CI [2.87-3.35] showing that for each increase in the rhinitis survey volume, headache research volume tends to increase by 3.11. Regarding migraine, for each increase in the rhinitis survey volume, there was an increase of 0.8 in the migraine survey volume, with a 95%CI [0.73-0.88].

**Figure 2:**
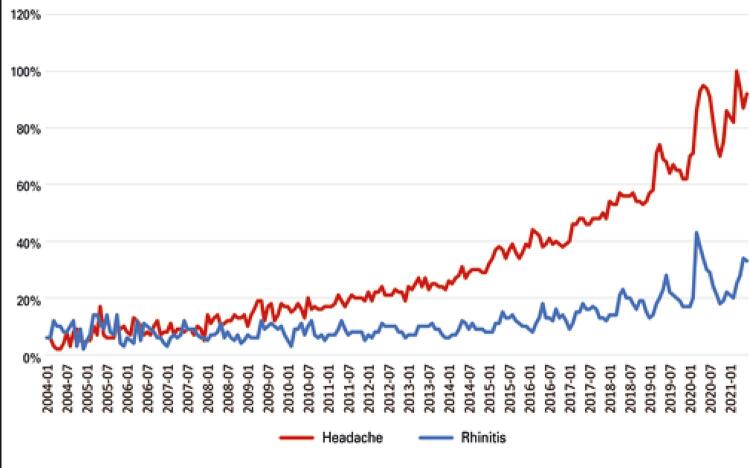
Comparison between headache volume search and rhinitis volume search on the Google Trends platform

Figure 2 shows the survey volume of the terms on the Google Trends platform. From the period of January 2004 to June 2021, a seasonal variation in the search for these terms is very clear, with peaks around month of May occurring every year. Additionally, from month to month, there appears to be a strong correlation between these two search queries. Most notably, however, is the fact that throughout this time period, the searches for rhinitis increased at a fraction of the rate that the searches for headaches did, which may suggest an increase in people experiencing headaches worldwide.

**Figure 3:**
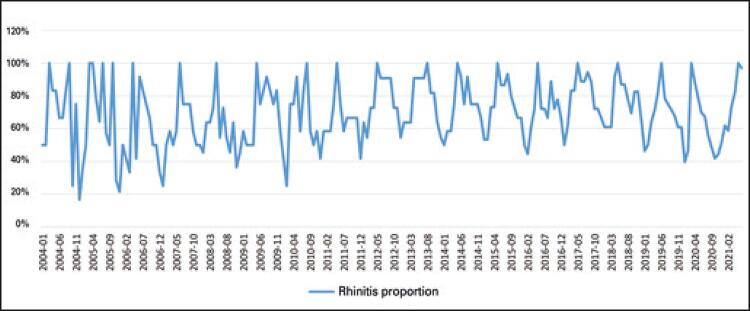
Seasonality of the search volume of the term rhinitis between 2004-2021, in the Google Trends platform

Figure 3 presents the seasonality of the search volume of the term rhinitis. The chart shows that the peaks of research volume tend to concentrate in the month of May, with the smaller volumes of research concentrating during the months of spring and early summer, that is, from October to February, and in the fall (months of May and June) this volume of research increases.

This result is similar to that found in a study of allergy prevalence with Google Trends in the United States and in parts of Europe, in which a peak was reported in April and May, seasonally, with a second lower peak occurring in September. Therefore, Google Trends has been reflecting the epidemiological behavior of allergic rhinitis.

**Figure 4:**
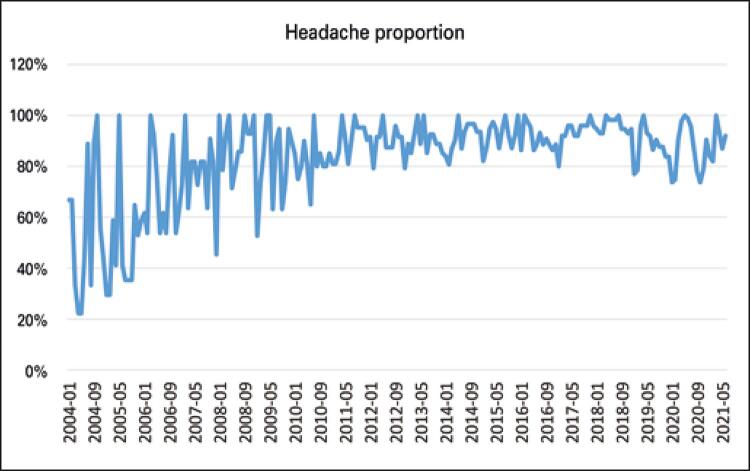
Seasonality of the search volume of the term headache between 2004-2021, in the Google Trends platform

Figure 4 shows that the distribution of the headache search volume has a pattern similar to the rhinitis volume, as expected, since the variables are strongly correlated. As in the rhinitis chart, the volume of search decreases in the months of the end of the year and beginning of the year, more precisely from November to January. On the other hand, the largest research volumes were concentrated in the months of April and August, presenting most of the peaks over the years.

## DISCUSSION

The present study showed that the volume of research on the terms headache and rhinitis has a strong correlation. In addition, this study found increased volume of search on the term headache and a seasonality in the search of the term rhinitis. Alzheimer’s was not correlated with the other terms.

These data suggest that in the fall months, the population is more likely to investigate rhinitis, which could indicate an increase in the prevalence of episodes of rhinitis during these months. Contrary to the study in Michigan,^(12)^ this study found decreased volume of rhinitis search in the spring and early summer months, which could indicate a lower prevalence of rhinitis at these times.

The association found between headaches and rhinitis may be explained by different aspects. First, allergic rhinitis might be causing headaches or worsening primary headaches. A study conducted in Poland found an overlap in migraine and allergic rhinitis symptoms, migraineurs worsened their headaches with exposure to allergens, and developed more nasal congestion and lacrimation.^(13)^ Migraine has long been connected to inflammation. Moskowitz^(14-16)^ proposed the neurovascular theory, observing protein plasma extravasation in the trigeminal structures. A rhinogenic underlying cause in migraine patients may be considered.

Allergies causing or worsening headaches is more accepted, but the other direction may also be hypothesized, although it is less likely to occur. Primary headaches may increase the chance of having allergic rhinitis, thus, comorbidity studies focusing on this topic should be done in order to confirm this hypothesis. Nevertheless, primary headaches with autonomic symptoms may be wrongly interpreted by patients as having rhinitis.

A third element may be causing both rhinitis and headaches, such as climate changes, temperature fluctuations, or even an internal, chronobiological dysfunction leading to more headaches and allergic symptoms. The hypothalamus and melatonin^(17,18)^ secretion could be involved to explain this mechanism.

The present study has some limitations. Google Trends is a database for all Google searches, which are made for a wide range of purposes, not just health related. This means that people may not be searching for these terms just because they are suffering from them. Furthermore, as explained previously, the search numbers reported are only relative numbers, and not actual number of searches. Also, the results of this study are heavily dependent on patients’ literacy, more specifically health literacy, as well as adequate access to the Internet, leading to a large population bias as to whose searches are represented in this study. This also highlights the fact that the number of searches for a term provides no information on the population demographics, just geographical information.

The research was limited to Portuguese speaking countries, since the objective of this study was to evaluate the Brazilian scenario with its particularities in climatic conditions, internet access, thus presenting a more specific and less universal result, limiting its reproducibility.

In the present study, the patient was self-diagnosed with one of the conditions, rhinitis or headache, which could lead to a diagnostic error by the patient, confusing the distribution of pathologies. This study shows through Google Trends, that these two terms surveyed by the public, “headache” and “migraine” are correlated with rhinitis, showing an understanding of the intersection between diseases by patients. Thus, during a clinical approach, this relation should be addressed, to complement the diagnostic investigation, not leading to overdiagnosed rhinitis, and to the underdiagnosis of primary headaches, since the study volumes are correlated through the study.

Another finding is the increase in relative search volume over time. This could suggest an increase in headache and rhinitis occurrence. Increase in headache relative search volume was higher than the one found in rhinitis. This may be due to a real increase in primary or secondary headache prevalence. Headache and rhinitis search volume fluctuated over the year, the fact that some technical, access related change in volume search could not be ruled out.

## CONCLUSION

Headaches and rhinitis were significantly correlated in 17 years of Google Search query data, where a circannual variation could be observed with both conditions. Relative search volume for headache increased over time. Further studies using digital search engine query data may be useful for better understanding of comorbidity in headache disorders and possible treatments.

## References

[1] 1. Nuti SV, Wayda B, Ranasinghe I, Wang S, Dreyer RP, Chen SI, et al. The use of google trends in health care research: a systematic review. PLoS One. 2014;9(10):e109583. 10.1371/journal.pone.0109583 PMC4215636 25337815

[2] 2. Carneiro HA, Mylonakis E. Google trends: a web-based tool for real-time surveillance of disease outbreaks. Clin Infect Dis. 2009;49(10):1557-64. Review. 10.1086/630200 19845471

[3] 3. Copeland P, Romano R, Zhang T, Hecht G, Zigmond D, Stefansen C. Google disease trends: an update. London: The International Society for Neglected Tropical; 2013 [cited 2013 Mar 3]. Available from: http://research.google.com/pubs/archive/41763.pdf

[4] 4. Ginsberg J, Mohebbi MH, Patel RS, Brammer L, Smolinski MS, Brilliant L. Detecting influenza epidemics using search engine query data. Nature. 2009;457(7232):1012-4. 10.1038/nature07634 19020500

[5] 5. Jun SP, Yoo HS, Choi S. Ten years of research change using Google Trends: from the perspective of big data utilizations and applications. Technol Forecast Soc Change. 2018;130:69-87. Review.

[6] 6. Gryglas A. Allergic rhinitis and chronic daily headaches: is there a link? Curr Neurol Neurosci Rep. 2016;16(4):33. Review. 10.1007/s11910-016-0631-z PMC4762930 26898685

[7] 7. Global Burden of Disease Study 2013 Collaborators. Global, regional, and national incidence, prevalence, and years lived with disability for 301 acute and chronic diseases and injuries in 188 countries, 1990-2013: a systematic analysis for the Global Burden of Disease Study 2013. Lancet. 2015;386(9995):743-800. Review. 10.1016/S0140-6736(15)60692-4 PMC4561509 26063472

[8] 8. Wise SK, Lin SY, Toskala E, Orlandi RR, Akdis CA, Alt JA, et al. International consensus statement on allergy and rhinology: allergic rhinitis. Int Forum Allergy Rhinol. 2018;8(2):108-352. 10.1002/alr.22073 PMC7286723 29438602

[9] 9. Dykewicz MS, Wallace DV, Baroody F, Bernstein J, Craig T, Finegold I, Huang F, Larenas-Linnemann D, Meltzer E, Steven G, Bernstein DI, Blessing-Moore J, Dinakar C, Greenhawt M, Horner CC, Khan DA, Lang D, Oppenheimer J, Portnoy JM, Randolph CR, Rank MA; Workgroup Chair and Cochair, Dykewicz MS, Wallace DV. Treatment of seasonal allergic rhinitis: an evidence-based focused 2017 guideline update. Ann Allergy Asthma Immunol. 2017;119(6):489-511.e41. 10.1016/j.anai.2017.08.012 29103802

[10] 10. Nathan RA. The burden of allergic rhinitis. Allergy Asthma Proc. 2007;28(1):3-9. Review. 10.2500/aap.2007.28.2934 17390749

[11] 11. Ceriani CE, Silberstein SD. Headache and rhinosinusitis: a review. Cephalalgia. 2021;41(4):453-63. Review. 10.1177/0333102420959790 32954817

[12] 12. Broder I, Barlow PP, Horton RJ. The epidemiology of asthma and hay fever in a total community, Tecumseh, Michigan. I. Description of study and general findings. J Allergy. 1962;33:513-23. 10.1016/0021-8707(62)90019-9 14015655

[13] 13. Patel ZM, Kennedy DW, Setzen M, Poetker DM, DelGaudio JM. “Sinus headache”: rhinogenic headache or migraine? An evidence-based guide to diagnosis and treatment. Int Forum Allergy Rhinol. 2013;3(3):221-30. Review. 10.1002/alr.21095 23129234

[14] 14. Moskowitz MA. The neurobiology of vascular head pain. Ann Neurol. 1984;16(2):157-68. 10.1002/ana.410160202 6206779

[15] 15. Moskowitz MA. Pathophysiology of headache--past and present. Headache. 2007;47 Suppl 1:S58-63. Review. 10.1111/j.1526-4610.2007.00678.x 17425711

[16] 16. Moskowitz MA, Reinhard JF Jr, Romero J, Melamed E, Pettibone DJ. Neurotransmitters and the fifth cranial nerve: is there a relation to the headache phase of migraine? Lancet. 1979;2(8148):883-5. 10.1016/s0140-6736(79)92692-8 90971

[17] 17. Gelfand AA, Goadsby PJ. The role of melatonin in the treatment of primary headache disorders. Headache. 2016;56(8):1257-66. Review. 10.1111/head.12862 PMC5012937 27316772

[18] 18. Bruera O, Sances G, Leston J, Levin G, Cristina S, Medina C, et al. Plasma melatonin pattern in chronic and episodic headaches: evaluation during sleep and waking. Funct Neurol. 2008;23(2):77-81 18671907

